# Mechanisms of Cisplatin-Induced Acute Kidney Injury: The Role of NRF2 in Mitochondrial Dysfunction and Metabolic Reprogramming

**DOI:** 10.3390/antiox14070775

**Published:** 2025-06-24

**Authors:** Jihan Liu, Yiming Wang, Panshuang Qiao, Yi Ying, Simei Lin, Feng Lu, Cai Gao, Min Li, Baoxue Yang, Hong Zhou

**Affiliations:** 1 Department of Pharmacology, School of Basic Medical Sciences, Peking University, Beijing 100191, China; liujihan@stu.pku.edu.cn (J.L.);; 2 Institute of Agri-Food Processing and Nutrition, Beijing Academy of Agriculture and Forestry Sciences, Beijing 100097, China

**Keywords:** acute kidney injury, cisplatin, NRF2, mitochondrial dysfunction, metabolic reprogramming

## Abstract

Cisplatin (Cis) is a widely used chemotherapy drug, but its nephrotoxicity limits its clinical application. Acute kidney injury (AKI) is a common complication, restricting long-term use. This study investigates the mechanisms of cisplatin-induced AKI and explores potential therapeutic targets. C57BL/6J mice were intraperitoneally injected with 20 mg/kg cisplatin to establish an AKI model. Serum creatinine, urea nitrogen, and tubular injury biomarkers (NGAL, KIM-1) progressively increased, indicating kidney dysfunction. Mitochondrial ATP levels significantly decreased, along with reduced mitochondrial fission and fusion, suggesting mitochondrial dysfunction. Increased oxidases and reduced antioxidants indicated redox imbalance, and metabolic reprogramming was observed, with lipid deposition, impaired fatty acid oxidation (FAO), and enhanced glycolysis in proximal tubular epithelial cells (PTECs). Nuclear factor erythroid 2-related factor 2 (NRF2) is a key transcriptional regulator of redox homeostasis and mitochondrial function. We found NRF2 levels increased early in AKI, followed by a decrease in vivo and in vitro, suggesting activation in the stress response. *Nfe2l2* knockout mice showed aggravated kidney injury, characterized by worsened kidney function and histopathological damage. Mechanistically, *Nfe2l2* knockout resulted in redox imbalance, reduced ATP synthesis, mitochondrial dysfunction and metabolic dysregulation. Furthermore, we activated NRF2 using dimethyl fumarate (DMF), observing a reduction in kidney damage and lipid deposition in mice. In conclusion, activating NRF2-dependent antioxidant pathways plays a crucial role in protecting against cisplatin-induced AKI. NRF2 may serve as a potential target for developing therapeutic strategies to prevent cisplatin nephrotoxicity.

## 1. Introduction

Cisplatin is a platinum-based chemotherapy drug widely used in the treatment of various malignancies and has been shown to significantly extend patient survival [1]. However, despite its remarkable anticancer efficacy, a common side effect—nephrotoxicity—limits its broader clinical application [2]. Studies have shown that approximately 25–35% of cisplatin-treated patients develop AKI, with the incidence of AKI increasing with higher doses. Preventive measures for cisplatin-induced AKI (Cis-AKI) include proper hydration and regular kidney function monitoring. Although these interventions can partially alleviate cisplatin nephrotoxicity, they are still insufficient to completely prevent AKI [3]. Therefore, effectively preventing and treating cisplatin-related AKI remains a significant clinical challenge.

The kidneys, as the primary organ responsible for the elimination of cisplatin, accumulate the drug predominantly in PTECs, which are the key sites for the initiation and progression of Cis-AKI. Cisplatin enters and accumulates in PTECs through passive diffusion or active transport, typically reaching concentrations more than five times higher than in the bloodstream, leading to tubular dysfunction and cumulative renal damage [4,5]. Within PTECs, cisplatin induces DNA damage, mitochondrial dysfunction, endoplasmic reticulum stress, oxidative stress, ferroptosis, autophagy, and cell cycle arrest, ultimately resulting in PTEC death, renal inflammation, and fibrosis, thereby accelerating the progression from Cis-AKI to chronic kidney disease (CKD) [6]. Although extensive research has identified several mechanisms underlying cisplatin nephrotoxicity, the precise molecular mechanisms remain incompletely understood [7,8].

Studies show that cisplatin damages both nuclear DNA (nDNA) and mitochondrial DNA (mtDNA), causing mitochondrial dysfunction in PTECs [9]. As mitochondria are crucial for energy production, their dysfunction leads to impaired cell metabolism and survival [10,11]. In response to ischemic and hypoxic stress, PTECs undergo metabolic reprogramming, with reduced FAO and increased glycolysis, to meet energy demands and maintain cell survival. Hypoxia-inducible factors (HIFs), especially HIF-1α, play a pivotal role in regulating this metabolic shift by promoting glycolytic gene expression and suppressing mitochondrial respiration [12]. However, this shift may disrupt cellular homeostasis, contributing to the progression of AKI and its transition to CKD [13,14]. The timing of metabolic reprogramming and FAO recovery after kidney injury remains unclear, and whether the early modulation of FAO can support kidney repair requires further investigation.

Oxidative stress is a key early event in Cis-AKI, and multiple studies have demonstrated the protective effects of antioxidant therapies in kidney diseases [15]. However, there are currently no approved antioxidant drugs specifically targeting renal injury. NRF2, a central transcription factor in the regulation of redox balance, orchestrates the expression of over 200 downstream genes involved in antioxidant defense, detoxification, inflammation suppression, and tissue repair [16,17]. Extensive preclinical and clinical evidence supports the renoprotective role of NRF2: animal studies have shown that NRF2 deficiency exacerbates injury in models of ischemia–reperfusion injury (IRI), unilateral ureteral obstruction (UUO), and hyperuricemic nephropathy, while its activation alleviates oxidative stress, inflammation, and fibrosis [18,19,20,21,22,23]. In human CKD patients, NRF2 protein levels correlate positively with renal function, and NRF2 activators such as bardoxolone methyl have demonstrated estimated GFR (eGFR) improvement in clinical trials, though safety concerns require careful patient selection [24,25,26]. However, its role beyond antioxidant defense in Cis-AKI remains unclear. Thus, exploring NRF2-dependent signaling in relation to energy metabolism and metabolic reprogramming in Cis-AKI is crucial.

This study first established a Cis-AKI model, revealing significant renal damage, mitochondrial dysfunction, and metabolic reprogramming. Through in vivo and in vitro experiments, we identified NRF2 as a crucial factor in maintaining mitochondrial function, redox homeostasis, and lipid metabolism. Our findings confirm that NRF2 plays a protective role in Cis-AKI and suggest that NRF2 may serve as a potential therapeutic target.

## 2. Materials and Methods

### 2.1. Animals

Eight-week-old male C57BL/6J mice (20–22 g) were purchased from the Experimental Animal Center of Peking University Health Science Center. *Nfe2l2* knockout mice (B6.129X1-*Nfe2l2^tm1Ywk^*/J) were obtained from Jackson Laboratory and were stably bred and maintained at the Animal Center of Peking University. Genotypes were confirmed by PCR, and adult male wild-type and *Nfe2l2* knockout mice (8–10 weeks old, 20–25 g) were selected for experiments.

All animals were housed in a specific pathogen-free facility under controlled conditions (22 °C, 50% humidity, 12/12-hour light/dark cycle) with ad libitum access to food and water. Mice were anesthetized with isoflurane and euthanized by cervical dislocation in accordance with institutional animal care guidelines. All animal care protocols were approved by the Institutional Animal Care and Use Committee at the Peking University Health Science Center. We followed the guidelines provided by China Association for Laboratory Animal Science for the care and use of laboratory animals (Ethics approval number: BCJB0060).

### 2.2. Cells

The immortalized human renal proximal tubule epithelial cell line, Human Kidney-2 (HK-2), was purchased from ATCC (catalog number CRL-2190). The cells were cultured in DMEM/F12 medium (03.2001C, EallBio, Beijing, China) supplemented with 10% fetal bovine serum (FBS), 100 μg/mL streptomycin, and 100 U/mL penicillin (03.17002DB, EallBio, Beijing, China), and maintained in a 37 °C incubator with 5% CO_2_. Cisplatin (HY-17394, MedChemExpress, Monmouth Junction, NJ, USA) was administered to cells at final concentrations of 20 and 40 µmol/L. The stock solution was diluted to the desired concentrations using a serum-free medium, and cells were treated for 24 h.

### 2.3. Animal Model Establishment and Drug Administration

Cis-AKI Model: Eight- to ten-week-old male C57BL/6J mice were randomly assigned to four groups: control and cisplatin groups (1-day, 2-day, and 3-day). Mice in the model groups received a single intraperitoneal injection of cisplatin (20 mg/kg), and samples were collected at 1-, 2-, and 3-days post-injection. The control group received an equal volume of sterile saline.

Cis-AKI in *Nfe2l2* Knockout (KO) Mice: KO mice and wild-type (WT) mice were divided into four groups: WT control, KO control, WT model, and KO model. The model groups received a single intraperitoneal injection of cisplatin (20 mg/kg), with sample collection on day 3. Control groups received an equal volume of saline.

Cisplatin-Induced Chronic Kidney Disease (Cis-CKD) Model: KO and WT mice were divided into four groups: WT control, KO control, WT model, and KO model. CKD was induced in model groups by intraperitoneal injection of cisplatin (5 mg/kg) once per week for four weeks. Samples were collected after the final injection. Control groups received saline.

NRF2 Activation in Cis-AKI Model: Mice were randomly assigned to control, model, and DMF treatment groups. DMF-treated mice received 10 mg/kg DMF (dissolved in 0.5% carboxymethyl cellulose sodium solution) via oral gavage 24 h before, on the day of, and 1- and 2-days post-cisplatin injection. The model group received cisplatin (20 mg/kg) intraperitoneally, and samples were collected on day 3. Control and model groups received an equal volume of vehicle solution. DMF (Dimethyl fumarate, HY-17363) was purchased from MCE (Monmouth Junction, NJ, USA).

Body weight was recorded daily (AKI models) or weekly (CKD model). On the collection day, blood and kidneys were harvested, and tissue weight was recorded. Blood was centrifuged (3000 rpm, 15 min, room temperature) to isolate the serum. All samples were stored at −80 °C.

### 2.4. Biochemical Analysis of Serum, Tissue, and Cell Samples

Serum creatinine (Scr) (C011-2-1, NJJC Bio, Nanjing, China), blood urea nitrogen (BUN) (C013-2-1, NJJC Bio, Nanjing, China), and xanthine oxidase (A002-1-1, NJJC Bio, Nanjing, China) levels were measured using commercial assay kits according to the manufacturer’s instructions. Adenosine triphosphate (ATP) levels in tissue and HK-2 cells, as well as lactate and lactate dehydrogenase (LDH) concentrations, were determined using assay kits from Solarbio (Beijing, China), following the manufacturer’s protocols. Triacylglycerol levels in tissues were measured using a commercial assay kit (E1013-50, Applygen, Beijing, China), according to the manufacturer’s instructions.

### 2.5. Measurement of Glomerular Filtration Rate (GFR) in Mice

On the day before measurement, the mice were anesthetized, and hair was removed from the right dorsal skin using depilatory cream. On the day of measurement, the mice were anesthetized with isoflurane, and a GFR monitor (MediBeacon, St. Louis, MO, USA) was attached to the exposed skin using adhesive patches and tape. After recovery from anesthesia, the mice were allowed free movement for 30 min to establish baseline levels.

Fluorescein isothiocyanate (FITC) tracer solution (MediBeacon, 7 mg/100 g) was administered via the tail vein, and GFR was measured over 1 h. For the mice with impaired renal function, the measurement duration was extended as needed. After the measurement, the device was connected to a dedicated software to calculate the GFR.

### 2.6. RNA Extraction and Quantitative RT-PCR

Mouse kidney tissues (~25 mg) stored at −80 °C were homogenized in 500 μL TRIgent solution (MF034-01, Mei5 Bio, Beijing, China) with beads. Total RNA was extracted using the chloroform–isopropanol method, washed with 75% ethanol, air-dried, and dissolved in DEPC-treated water. For cultured cells, RNA was extracted similarly after adding TRIgent and detaching cells by pipetting. RNA concentration and purity were measured using a NanoDrop. cDNA was synthesized using a reverse transcription kit (R333-01, Vazyme, Nanjing, China), following the manufacturer’s instructions. qPCR was performed with SYBR qPCR Mix (Q712-02, Vazyme, Nanjing, China) on a QuantStudio^®^ 3 system. RNA expression levels were calculated based on the CT value. Primer sequences are provided in Table A1.

### 2.7. Mouse Kidney Embedding and Sectioning

For paraffin embedding, mouse kidneys were fixed in 4% paraformaldehyde (PFA) overnight, dehydrated through graded ethanol, cleared in xylene:butanol (1:1), and embedded in paraffin. Sections (4 μm) were prepared using a microtome and baked at 60 °C before staining.

For frozen sections, kidneys were fixed in 4% PFA, cryoprotected in 20% and then 30% sucrose, embedded in Tissue Freezing Medium (14020108926, Leica, Wetzlar, Germany), and stored at −80 °C. Sections (8 μm) were cut using a cryostat and air-dried before staining or storage at −20 °C.

### 2.8. Hematoxylin and Eosin (H&E) Staining

Paraffin sections were baked at 60 °C for 10–15 min, deparaffinized in xylene twice for 10 min each, and rehydrated through 100%, 100%, 95%, 90%, 80%, and 70% graded ethanol for 3 min each, followed by a distilled water rinse. Sections were stained with Cole hematoxylin (G1140, Solarbio, Beijing, China) for 8 min, rinsed with 1% acid ethanol, and blued in 1% ammonia water. After eosin (ZLI-9613, ZSGB-BIOMF, Beijing, China) staining for 5 s, sections were dehydrated in graded ethanol, cleared in xylene for 2 min, and mounted with neutral resin. After drying, sections were scanned using a slide scanner.

To ensure objectivity and minimize bias, the histological examinations of the kidney were performed by an investigator who was blinded to the group allocation. Renal tubular injury was scored using the Paller method [27], where tubular dilation and cell flattening were assigned 1 point, brush border damage 1 point, brush border loss 2 points, tubular casts 2 points, and the presence of necrotic or detached tubular cells 1 point.

### 2.9. Masson’s Trichrome Staining

Paraffin sections were deparaffinized and rehydrated to distilled water. The sections were stained with picric acid–fuchsin solution for 5–10 min, washed with a weak acid solution (distilled water: weak acid = 2:1) for 30 s, and treated with phosphomolybdic acid for 1–2 min. After another weak acid wash (30 s), the sections were stained with aniline blue for 1–2 min. Subsequent steps included another weak acid wash (30 s), rapid dehydration in 95% ethanol (2–3 s), dehydration in absolute ethanol twice (5–10 s each), clearing in xylene twice (1–2 min each), and mounting with neutral resin. After drying, the slides were scanned using a slide scanner. The Masson’s Trichrome Staining Kit was purchased from Solarbio, Beijing, China.

### 2.10. Oil Red O Staining

The frozen sections were washed three times with PBS (5 min each) and immersed in 60% isopropanol for 10 min. Meanwhile, the Oil Red O stock solution was prepared by dissolving 0.25 g Oil Red O powder (O9755, Sigma-Aldrich, St. Louis, MO, USA) in 50 mL isopropanol. The working solution was prepared by mixing Oil Red O stock with distilled water at a 3:2 ratio. The sections were stained in Oil Red O working solution for 10 min, followed by differentiation in 60% isopropanol. After nuclear staining with Cole hematoxylin for 8 min and a distilled water rinse, slides were mounted with glycerin gelatin and scanned using a slide scanner.

### 2.11. Western Blot Analysis

Total renal protein was extracted by homogenizing 20 mg of mouse kidney tissue in 200 μL lysis buffer (RIPA lysis buffer: phosphatase inhibitor: protease inhibitor = 98:1:1). Nuclear protein was extracted using a nuclear protein extraction kit (P1202-50, Applygen, Beijing, China), following the manufacturer’s instructions.

Protein concentration was determined using the BCA method (ZJ102, Epizyme, Shanghai, China), and samples were mixed with RIPA buffer and loading buffer, then denatured at 100 °C for 10 min (or at room temperature for 30 min for oxidative phosphorylation proteins), cooled on ice, and stored at −80 °C if not used immediately. SDS-PAGE was conducted with an appropriate gel concentration, and the samples (20–30 µg) were loaded alongside protein markers. Electrophoresis was run at 80 V for stacking and 120 V for separation. After methanol activation, PVDF membranes (IPVH00010, Millipore, Burlington, MA, USA) were used for protein transfer at 200 mA in an ice bath for 2 h, with extended time for high-molecular-weight proteins. Membranes were blocked with 5% skim milk in TBST at room temperature for 2 h, and then incubated with primary antibodies overnight at 4 °C. After TBST washes, membranes were incubated with HRP-conjugated secondary antibodies at room temperature for 45 min, followed by further washes. Protein bands were detected using ECL chemiluminescence (RM02867, ABclonal, Wuhan, China) and analyzed with ImageJ 1.54g. The antibodies used for the Western blot are listed in Table A2.

### 2.12. Immunohistochemistry

Paraffin sections were deparaffinized and incubated with 3% hydrogen peroxide for 10 min. After washing with PBS, antigen retrieval (C1038, Solarbio, Beijing, China) was performed in a boiling water bath for 15 min, followed by cooling at room temperature. The sections were washed with PBS, and for nuclear staining, 0.3% TritonX-100 (T6200, Biotopped, Beijing, China) was applied for 20 min. After blocking with 5% goat serum (MF476-01, Mei5 Bio, Beijing, China) for 1 h, the primary antibody was applied and incubated overnight at 4 °C. The next day, the secondary antibody was applied for 1 h at room temperature, followed by DAB staining (ZLI-9018, ZSGB-BIOMF, Beijing, China). After hematoxylin counterstaining, the slides were dehydrated, cleared in xylene, mounted, and scanned. The antibodies used are listed in Table A2.

### 2.13. Immunofluorescence

HK-2 cells were seeded in black glass-bottom 96-well plates (P96-1.5H-N, Cellvis, Mountain View, CA, USA) and allowed to adhere. After stimulation, the cells were fixed with 4% paraformaldehyde for 20 min, washed with PBS, and permeabilized with 0.3% TritonX-100 for 20 min. After blocking with 5% goat serum for 1 h, the primary antibody was added and incubated overnight at 4 °C. The next day, the secondary antibody was added and incubated at room temperature for 45 min. The cells were stained with DAPI (C0060, Solarbio, Beijing, China) for 10 min, washed, and images were captured using a high-content analysis system. The antibodies used are listed in Table A2.

### 2.14. Cell Viability Assay

HK-2 cells were seeded in a 96-well plate and cultured until they adhered and reached about 80% confluence. Different concentrations of cisplatin were added to the wells and incubated for 24 h. A CCK-8 (03.17002DB, EallBio, Beijing, China) working solution was prepared by mixing serum-free medium with CCK-8 solution at a 100:10 ratio. After removing the original culture medium, cells were washed with PBS three times, and 100 µL of the CCK-8 working solution was added to each well. The plate was incubated for 1 h, and absorbance was measured at 450 nm using a microplate reader.

### 2.15. Mitochondrial Membrane Potential Assay

HK-2 cells were seeded in a 12-well plate and incubated until 80% confluence. Different concentrations of cisplatin were added and incubated for 24 h. After stimulation, cells were washed with PBS and digested with trypsin. They were then resuspended in 0.5 mL cell culture medium and stained with 0.5 mL JC-1 working solution (M8650, Solarbio, Beijing, China). After incubation at 37 °C for 20 min, the cells were washed twice with JC-1 staining buffer and centrifuged at 800× *g* for 3 min. The cells were then resuspended in JC-1 staining buffer, passed through a 40 μm filter, and analyzed by flow cytometry.

### 2.16. Reactive Oxygen Species (ROS) Staining

HK-2 cells were seeded in a 12-well plate and incubated until 80% confluence. Cisplatin was added and incubated for varying times. After removing the culture medium, the cells were washed with PBS and stained with DCFH-DA probe (diluted in serum-free medium, 1:1500, Applygen, Beijing, China). The positive control samples were treated with both DCFH-DA and 50 µM ROS donor. After 30 min incubation at 37 °C, the excess dye was washed off using serum-free medium. The cells were then stained with Hoechst solution for 20 min, and the excess dye was removed. Finally, the ROS levels were detected using a fluorescence microscope under FITC conditions.

### 2.17. Bioinformatics Analysis of Kidney RNA Sequencing

The RNA sequencing data used in this study were obtained from the Gene Expression Omnibus (GEO) database (https://www.ncbi.nlm.nih.gov/geo/, accessed on 13 February 2025), with data ID: GSE207587 [28]. Raw sequencing files were downloaded from GEO, and analyses were performed comparing the control and cisplatin model groups. Differential genes were identified using the R 4.4.2 software, selecting genes with a logFC greater than 1 or less than −1 and an adjusted *p*-value less than 0.05. Volcano plots and heatmaps were generated to visualize the differential expression of genes.

Differential genes were imported into the David database [29,30] (https://davidbioinformatics.nih.gov/, accessed on 13 February 2025) for Kyoto Encyclopedia of Genes and Genomes (KEGG) and Gene Ontology (GO) analyses. The top 10 related pathways were selected, and a bubble plot was created using the bioinformatics platform (https://www.bioinformatics.com.cn/, accessed on 13 February 2025).

The NRF2 target gene set was constructed using databases such as WikiPathway and KEGG. The overlap between NRF2 target genes and the identified differential genes was determined using Venny 2.1 (https://bioinfogp.cnb.csic.es/tools/venny/, accessed on 13 February 2025). The expression levels of NRF2 downstream genes (*Nqo1*, *Sod3*, *Gclc*, *Txnrd1*, *Ppargc1a*, and *Hsp90aa1*) were calculated based on the average expression levels of each gene, and their ratios were computed.

### 2.18. Statistical Analysis

All data in this study were analyzed using GraphPad Prism 10 software and are presented as mean ± standard error of the mean (mean ± SEM). Statistical analyses were performed using two-tailed Student’s *t*-test for comparisons between two groups or a one-way ANOVA with Tukey’s multiple comparison tests for comparisons among multiple groups. For datasets that did not follow a normal distribution, or that remained non-normally distributed after log-transformation, a non-parametric one-way ANOVA (Kruskal–Wallis test) was applied. A *p*-value < 0.05 was considered statistically significant. All in vitro and in vivo experiments were repeated at least three times.

## 3. Results

### 3.1. Progressive Kidney Function and Structural Damage in Cis-AKI Mice

**Figure 1:**
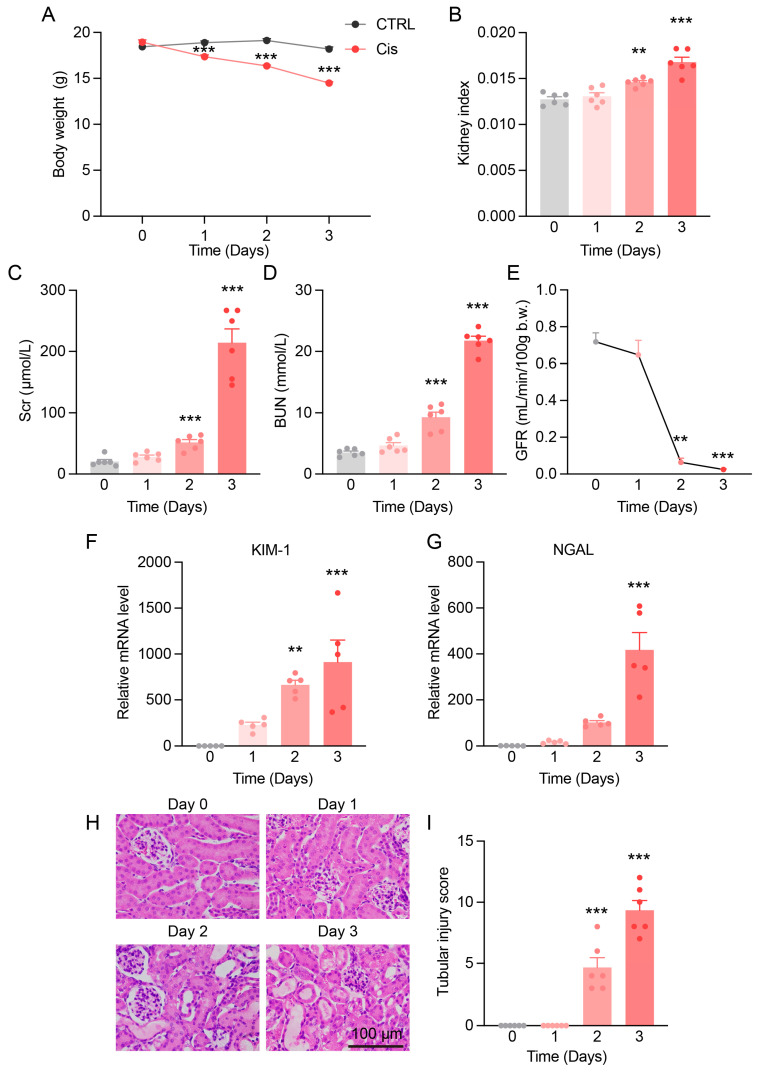
Cis-AKI mice show progressive decline in kidney function. (**A**,**B**) Body weight and kidney index (kidney weight/body weight) of mice at different time points post-cisplatin injection. *n* = 6. (**C**,**D**) Changes in Scr and BUN levels over time. *n* = 6. (**E**) GFR in Cis-AKI mice. *n* = 3–10. (**F**,**G**) Relative mRNA expression levels of KIM-1 and NGAL in the kidney cortex of Cis-AKI mice. *n* = 5. (**H**) H&E staining of mouse kidney cortex, scale bar = 100 µm. (**I**) Quantification of tubular injury score in mouse kidney sections. *n* = 6. Data are presented as means ± SEM. ** *p* < 0.01 and *** *p* < 0.001 vs. day 0.

Cis-AKI mice exhibited progressive weight loss and a significant increase in kidney index by day 3, indicating renal injury (Figure 1A,B). Kidney function was severely impaired, with Scr and BUN levels markedly elevated (Figure 1C,D). GFR decreased significantly by day 2 and was nearly abolished by day 3 (Figure 1E), reflecting rapid renal dysfunction.

The expression of proximal tubular injury markers showed that KIM-1 increased sharply from day 1 and continued rising, while NGAL exhibited a gradual but sustained elevation (Figure 1F,G). H&E staining showed normal kidney morphology in control mice, tubular injury, and cast formation by day 2 and pronounced damage by day 3 (Figure 1H,I).

**Figure A1:**
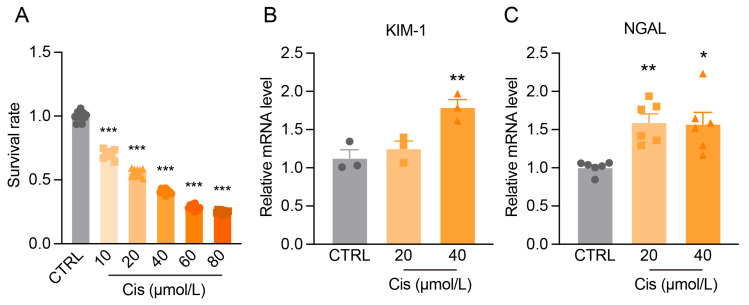
Cisplatin induces injury in HK-2 cells. (**A**) Cell viability of HK-2 cells treated with various concentrations of cisplatin for 24 h. n = 10. (**B**,**C**) Relative mRNA expression levels of KIM-1 and NGAL in HK-2 cells following treatment with different concentrations of cisplatin. Data are presented as means ± SEM. * *p* < 0.05, ** *p*< 0.01 and *** *p*< 0.001 vs. CTRL group.

To validate the PTEC injury model in vitro, HK-2 cells were treated with increasing doses of cisplatin, resulting in a dose-dependent decline in viability (Figure A1A) and upregulation of KIM-1 and NGAL (Figure A1B,C), confirming cisplatin-induced cytotoxicity in PTECs.

### 3.2. Mitochondrial Dysfunction and Redox Imbalance in the Kidneys of Cis-AKI Mice

To further investigate mitochondrial dysfunction and redox imbalance in Cis-AKI, we assessed mitochondrial homeostasis and oxidative stress markers.

**Figure 2:**
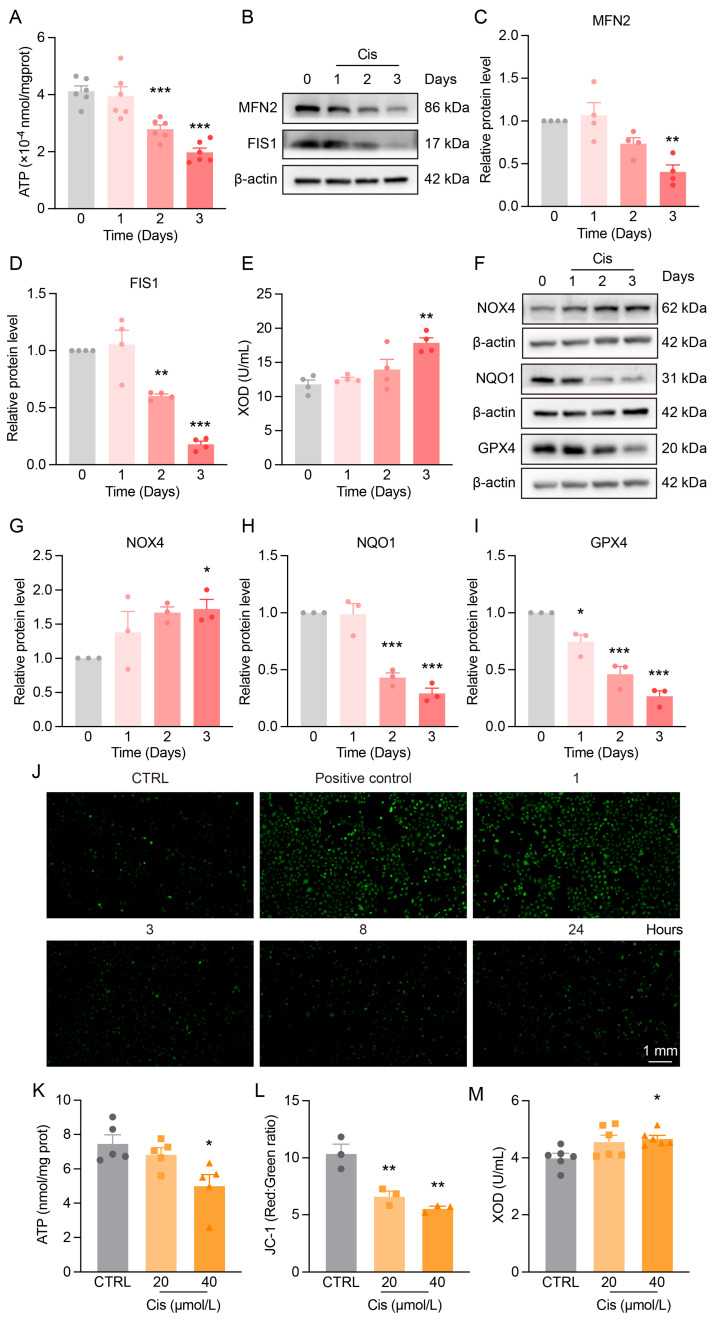
Cis-AKI mice and HK-2 cells exhibit mitochondrial dynamic imbalance and redox homeostasis disruption. (**A**) Changes in ATP levels in the renal cortex of Cis-AKI mice. *n* = 6. (**B**) Representative Western blot of fusion and fission proteins MFN2 and FIS1 in mouse kidneys. (**C**,**D**) Relative protein expression levels of MFN2 and FIS1 in mouse kidneys. *n* = 4. (**E**) Changes in serum XOD levels in Cis-AKI mice. *n* = 4. (**F**) Representative Western blot of antioxidant and oxidase enzymes NOX4, NQO1, and GPX4 in mouse kidneys. (**G**–**I**) Relative protein expression levels of NOX4, NQO1, and GPX4 in mouse kidneys. *n* = 3. Data are presented as means ± SEM. * *p* < 0.05, ** *p* < 0.01 and *** *p* < 0.001 vs. day 0. (**J**) ROS staining (green) in HK-2 cells after treatment with 40 μmol/L cisplatin for different time periods, scale bar = 1 mm. (**K**) Intracellular ATP levels in HK-2 cells after 24-hour treatment with varying concentrations of cisplatin. *n* = 5. (**L**) Ratio of JC-1 aggregates to JC-1 monomers in HK-2 cells after 24-hour treatment with different concentrations of cisplatin. *n* = 3. (**M**) XOD levels in HK-2 cells following 24-hour exposure to various concentrations of cisplatin. *n* = 6. Data are presented as means ± SEM. * *p* < 0.05 and ** *p* < 0.01 vs. CTRL.

ATP levels in the renal cortex of Cis-AKI mice significantly decreased on day 2 and further declined by day 3 (Figure 2A). The mitochondrial fusion protein MFN2 was reduced on day 3, and the fission protein FIS1 was downregulated on both day 2 and day 3 (Figure 2B,D), indicating disrupted mitochondrial dynamics.

Cisplatin treatment also enhanced oxidative stress. XOD and NOX4 levels were significantly elevated (Figure 2E–G), while antioxidant enzymes NQO1 and GPX4 were markedly decreased (Figure 2F,H,I), reflecting impaired redox balance and antioxidant defenses.

In vitro, cisplatin increased ROS production after 1 h (Figure 2J), decreased intracellular ATP levels (Figure 2K), elevated mitochondrial membrane potential (Figure 2L and Figure A3), and upregulated XOD (Figure 2M), consistent with in vivo findings.

### 3.3. Metabolic Reprogramming in the Kidneys of Cis-AKI Mice

To further explore the impact of Cis-AKI on renal energy metabolism, we assessed alterations in lipid and glucose metabolic pathways.

**Figure 3:**
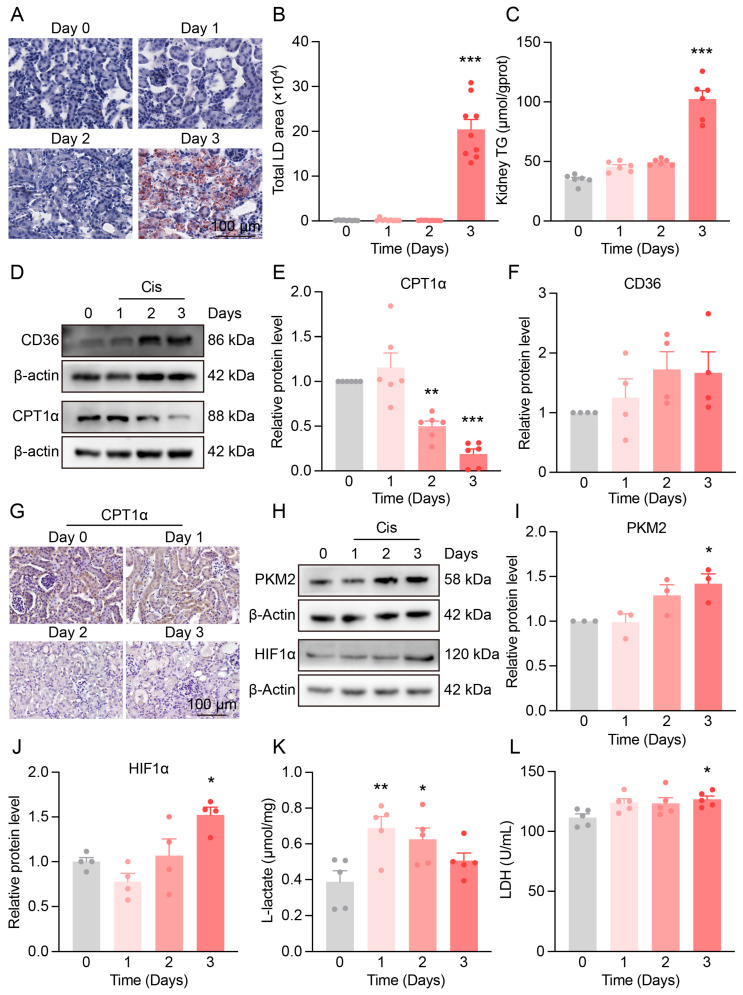
Metabolic reprogramming in the kidneys of Cis-AKI mice. (**A**) Representative Oil Red O staining images of kidney cortex in Cis-AKI mice, scale bar = 100 µm. (**B**) Quantification of lipid droplet area from Oil Red O staining. *n* = 9. (**C**) Triglyceride (TG) levels in the kidney cortex of Cis-AKI mice. *n* = 6. (**D**) Representative Western blot of CD36 and CPT1α expression in mouse kidneys. (**E**,**F**) Relative protein expression levels of CPT1α (*n* = 6) and CD36 (*n* = 4) in mouse kidneys. (**G**) Representative immunohistochemistry images of CPT1α in the kidney cortex of mice, scale bar = 100 µm. (**H**) Representative Western blot of PKM2 and HIF-1α expression in mouse kidneys. (**I**,**J**) Relative protein expression levels of PKM2 (*n* = 3) and HIF-1α (*n* = 4) in mouse kidneys. (**K**) Lactate levels in the kidneys of mice. *n* = 5. (**L**) L-lactate dehydrogenase levels in the serum of mice. *n* = 5. Data are presented as means ± SEM. * *p* < 0.05, ** *p* < 0.01 and *** *p* < 0.001 vs. day 0.

Three days after cisplatin administration, Oil Red O staining showed a significant accumulation of lipid droplets in renal tubules (Figure 3A,B), accompanied by increased triglyceride levels (Figure 3C), indicating impaired lipid utilization. Western blot analysis revealed the upregulation of CD36, a fatty acid transporter, and the downregulation of CPT1α, a key enzyme in FAO (Figure 3D–G), suggesting that lipid accumulation may result from enhanced fatty acid uptake and suppressed FAO.

Given the high energy demand of injured proximal tubules and their susceptibility to hypoxia, we next examined glycolytic activity. PKM2 expression was significantly increased on day 3 post-cisplatin treatment (Figure 3H,I), along with elevated HIF-1α levels (Figure 3H,J), indicating the activation of a hypoxia-driven metabolic response. Additionally, lactate levels rose rapidly following cisplatin administration, and LDH activity increased at later stages (Figure 3K–L), consistent with enhanced glycolytic flux.

### 3.4. Changes in NRF2 Expression and Localization After Cisplatin Treatment

**Figure 4:**
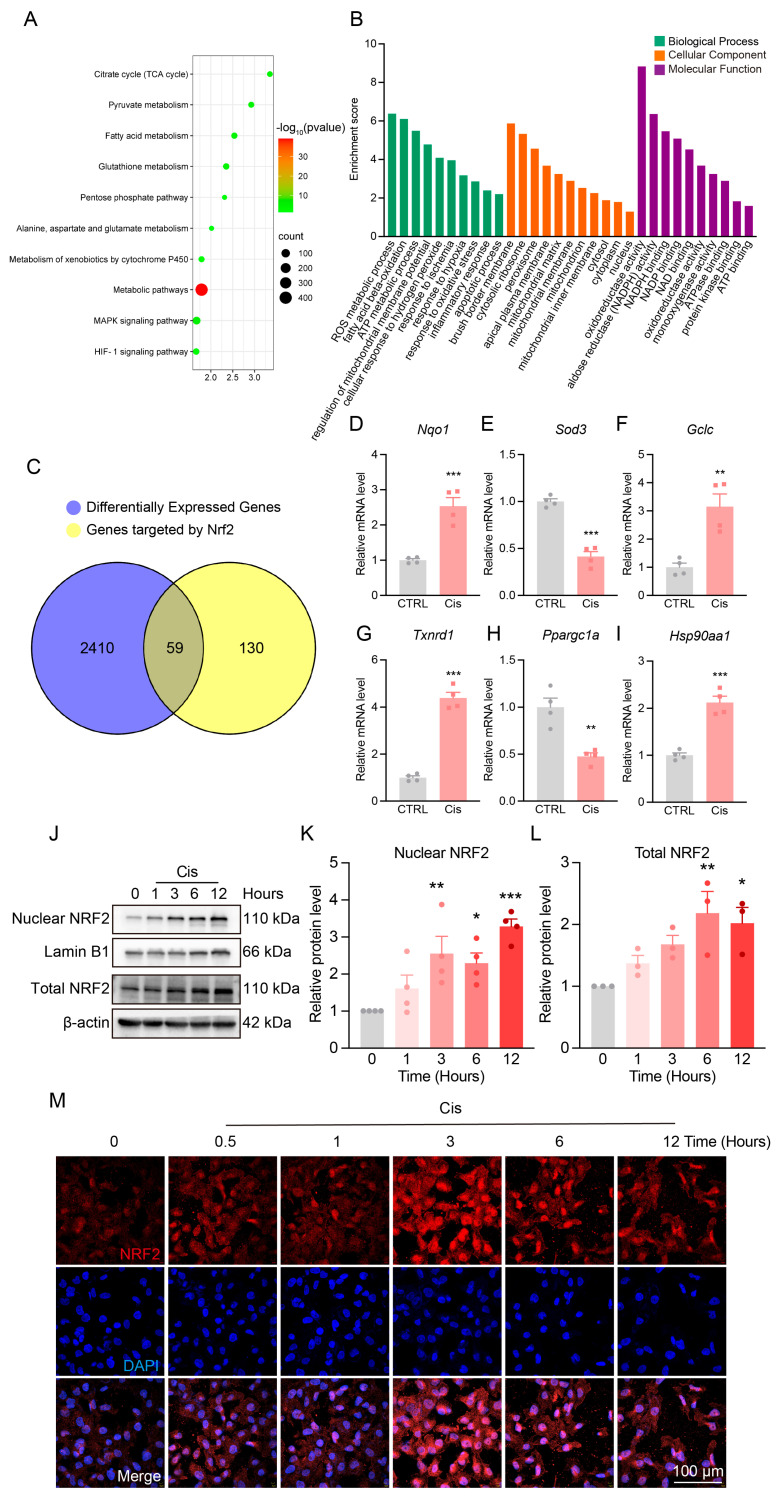
Changes in NRF2 expression and localization after cisplatin treatment in vivo and in vitro. (**A**) Bubble plot of KEGG pathway analysis for differentially expressed genes. (**B**) GO enrichment analysis of differentially expressed genes, with green representing biological process (BP), orange representing cellular component (CC), and purple representing molecular function (MF). (**C**) Venn diagram showing the overlap between differentially expressed genes and NRF2 downstream target genes. (**D**–**I**) Relative expression levels of NRF2 downstream genes *Nqo1*, *Sod3*, *Gclc*, *Txnrd1*, *Ppargc1a*, and *Hsp90aa1* from RNA sequencing data. *n* = 4. Data are presented as means ± SEM. ** *p* < 0.01 and *** *p* < 0.001 vs. CTRL. (**J**) Representative Western blot showing total and nuclear NRF2 protein levels in the kidneys of Cis-AKI mice at early time points (1, 3, 6, and 12 h) after cisplatin treatment. (**K**,**L**) Quantification of nuclear (*n* = 4) and total (*n* = 3) NRF2 protein levels in the kidneys of Cis-AKI mice at early time points after cisplatin treatment. (**M**) Immunofluorescence images of NRF2 (red) in HK-2 cells treated with cisplatin for different durations. Nuclei are stained with DAPI (blue), scale bar = 100 µm. Data are presented as means ± SEM. * *p* < 0.05, ** *p* < 0.01, and *** *p* < 0.001 vs. hour 0.

**Figure A2:**
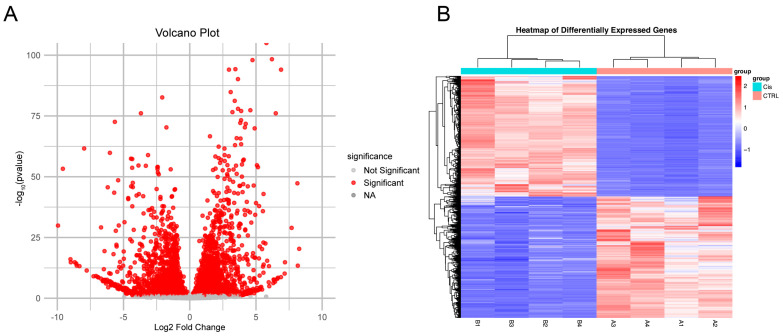
Cisplatin induces transcriptional changes in mouse kidneys. (**A**) Volcano plot of differentially expressed genes; red dots represent significantly altered genes. (**B**) Heatmap of differentially expressed genes.

**Figure A3:**
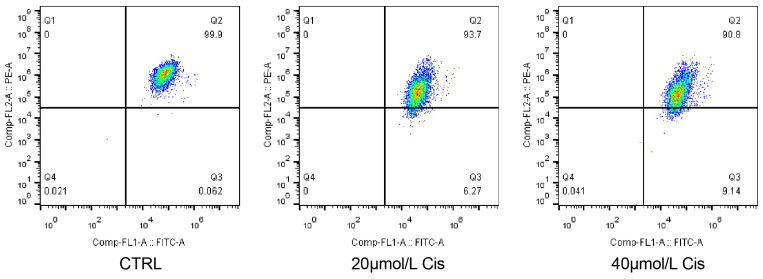
Representative flow cytometry plots of mitochondrial membrane potential.

To elucidate key molecular mechanisms of Cis-AKI and identify potential therapeutic targets, we performed transcriptomic analysis using RNA-seq data from cisplatin-treated mouse kidneys (Figure A2A,B). A total of 2469 differentially expressed genes were identified. The KEGG pathway analysis revealed significant enrichment in metabolic pathways, including the TCA cycle and fatty acid metabolism (Figure 4A). The GO analysis showed these differentially expressed genes were predominantly involved in biological processes related to ROS and ATP metabolism, highlighting oxidative stress as a central feature of Cis-AKI (Figure 4B).

Given these findings, we focused on NRF2, a redox-sensitive transcription factor that regulates antioxidant and metabolic gene expression. The intersection analysis revealed 59 differentially expressed genes overlapped with known NRF2 targets, accounting for 31% of its downstream genes (Figure 4C). Several classical NRF2-regulated genes—including *Nqo1*, *Sod3*, *Gclc*, *Txnrd1*, *Ppargc1a*, and *Hsp90aa1*—were significantly altered following cisplatin treatment, suggesting NRF2 pathway activation (Figure 4D–I).

To further validate NRF2 activation, we assessed its protein expression and nuclear localization in vivo. NRF2 levels progressively increased within 12 h of post-cisplatin exposure (Figure 4J–L). Similarly, in HK-2 cells, NRF2 expression and nuclear translocation peaked at 3 h post-treatment and declined thereafter (Figure 4M), indicating early activation followed by suppression during sustained stress.

### 3.5. Loss of NRF2 Aggravates Cisplatin-Induced Renal Dysfunction and Structural Damage

**Figure 5:**
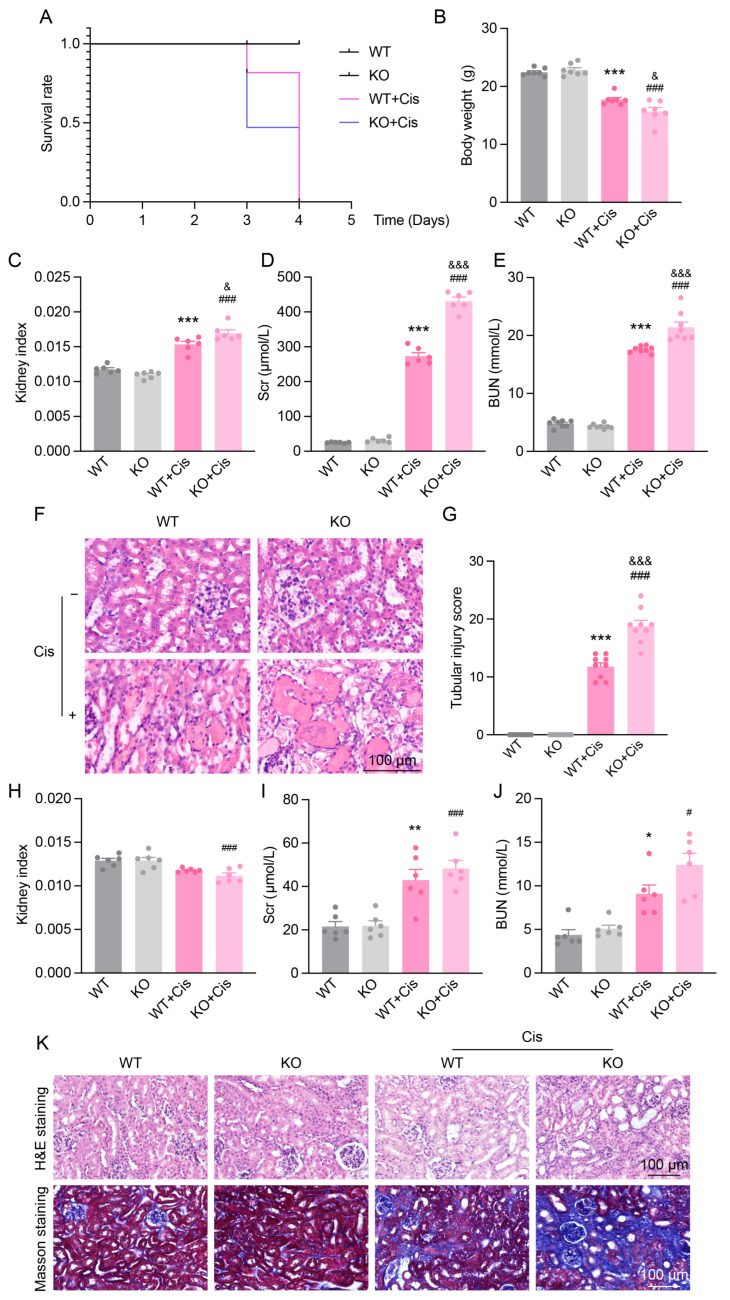
Renal function and structural changes in *Nfe2l2* gene knockout mice in AKI and CKD models. (**A**) Survival rate of WT and *Nfe2l2* gene KO mice after cisplatin administration. (**B**,**C**) Body weight (*n* = 7) and kidney index (kidney weight/body weight, *n* = 6) of different genotypes at day 3 after cisplatin administration. (**D**,**E**) Scr (*n* = 6) and BUN (*n* = 8) levels in different genotypes at day 3 after cisplatin administration. (**F**,**G**) Representative H&E staining images and tubular injury scores of different genotypes at day 3 after cisplatin administration, scale bar = 100 µm. *n* = 9. (**H**–**J**) Changes in kidney index, Scr, and BUN levels in KO and WT mice after 4 weeks of cisplatin treatment in the Cis-CKD model. *n* = 6. (**K**) Representative H&E and Masson’s trichrome staining images of KO and WT mice after 4 weeks of cisplatin treatment in the Cis-CKD model, scale bar = 100 µm. Data are presented as means ± SEM. * *p* < 0.05, ** *p* < 0.01 and *** *p* < 0.001 vs. WT group. ^#^ *p* < 0.05 and ^###^ *p* < 0.001 vs. KO group. ^&^ *p* < 0.05 and ^&&&^ *p* < 0.001 vs. WT + Cis group.

To further validate the role of NRF2 in Cis-AKI, we generated systemic *Nfe2l2* knockout mice and compared their responses to WT mice following cisplatin treatment. By day 3, KO mice exhibited significantly higher mortality (50%) than WT mice (Figure 5A), accompanied by greater weight loss and increased kidney index (Figure 5B,C). Renal function was more severely impaired in KO mice, as evidenced by elevated Scr and BUN levels (Figure 5D,E). H&E staining revealed exacerbated tubular damage, including more extensive brush border loss and cast formation in KO kidneys (Figure 5F,G), indicating that NRF2 deficiency worsens acute renal injury.

To assess chronic effects, we employed a low-dose, repeated cisplatin regimen. KO mice showed a more pronounced decrease in kidney index (Figure 5H) and higher—but not statistically significant—Scr and BUN levels compared to WT mice (Figure 5I,J). The histological examination revealed more severe tubular injury and collagen deposition in KO mice, as shown by enhanced fibrotic staining in Masson’s trichrome (Figure 5K), suggesting that NRF2 deficiency aggravates renal fibrosis under chronic cisplatin exposure.

### 3.6. Loss of NRF2 Aggravates Cisplatin-Induced Renal Mitochondrial Dysfunction

**Figure 6:**
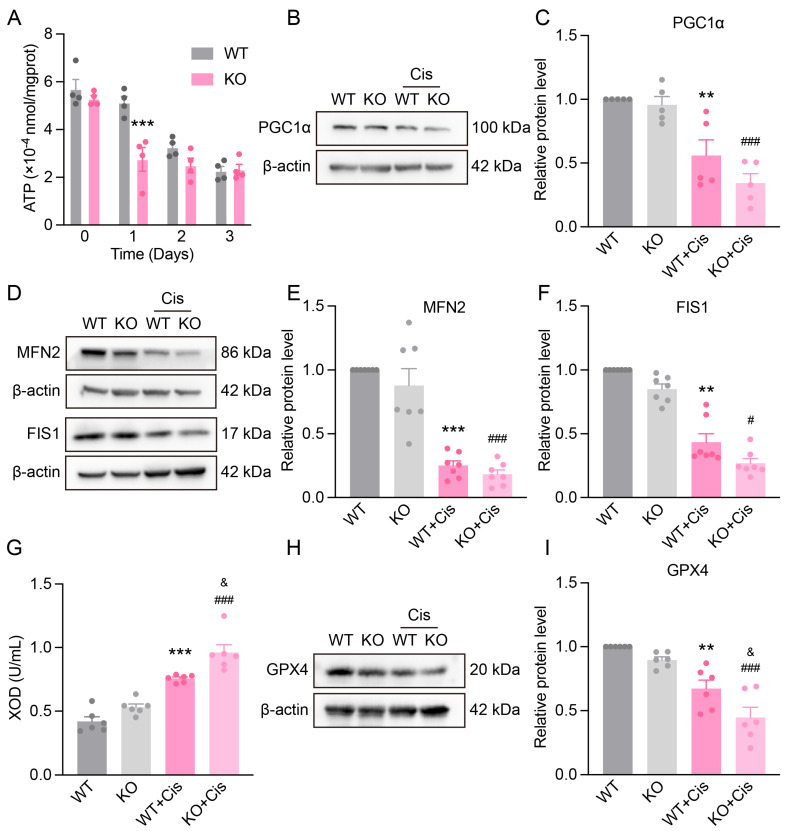
The impact of *Nfe2l2* gene knockout on cisplatin-induced mitochondrial dysfunction. (**A**) ATP levels in the renal cortex of WT and KO mice at different time points (1, 2, 3 days) after cisplatin administration. *n* = 4. (**B**) Representative Western blot images of PGC1α in the kidneys of different genotype mice after cisplatin treatment. (**C**) Relative protein expression levels of PGC1α. *n* = 5. (**D**) Representative Western blot images of MFN2 and FIS1 in the kidneys of different genotype mice after cisplatin treatment. (**E**,**F**) Relative protein expression levels of MFN2 and FIS1. *n* = 7. (**G**) Serum XOD levels in different genotype mice after cisplatin treatment. *n* = 6. (**H**) Representative Western blot images of GPX4 in the kidneys of different genotype mice after cisplatin treatment. (**I**) Relative protein expression levels of GPX4. *n* = 6. Data are presented as means ± SEM. ** *p* < 0.01 and *** *p* < 0.001 vs. WT group. ^#^ *p* < 0.05 and ^###^ *p*< 0.001 vs. KO group. ^&^ *p* < 0.05 vs. WT + Cis group.

We examined the role of NRF2 in mitochondrial function after cisplatin treatment. ATP levels in both WT and KO mice gradually decreased over time; however, the ATP content in KO kidneys was significantly lower than WT at day 1 post-injury (Figure 6A). PGC1α, a key transcriptional coactivator for mitochondrial biogenesis, showed a more pronounced decline in KO mice following cisplatin exposure (Figure 6B,C), indicating NRF2’s involvement in cisplatin-induced metabolic alterations.

Considering mitochondrial dynamics, cisplatin significantly reduced mitochondrial fusion protein MFN2 and fission protein FIS1 levels in the renal cortex, with greater reductions observed in KO mice (Figure 6D–F). Furthermore, KO mice exhibited a more marked increase in serum XOD and a stronger decrease in renal GPX4 levels after cisplatin treatment (Figure 6G–I). These findings suggest NRF2 mitigates cisplatin-induced oxidative stress by regulating antioxidant enzymes, and its deficiency exacerbates renal susceptibility to injury.

### 3.7. Loss of NRF2 Aggravates Cisplatin-Induced Metabolism Disruption

**Figure 7:**
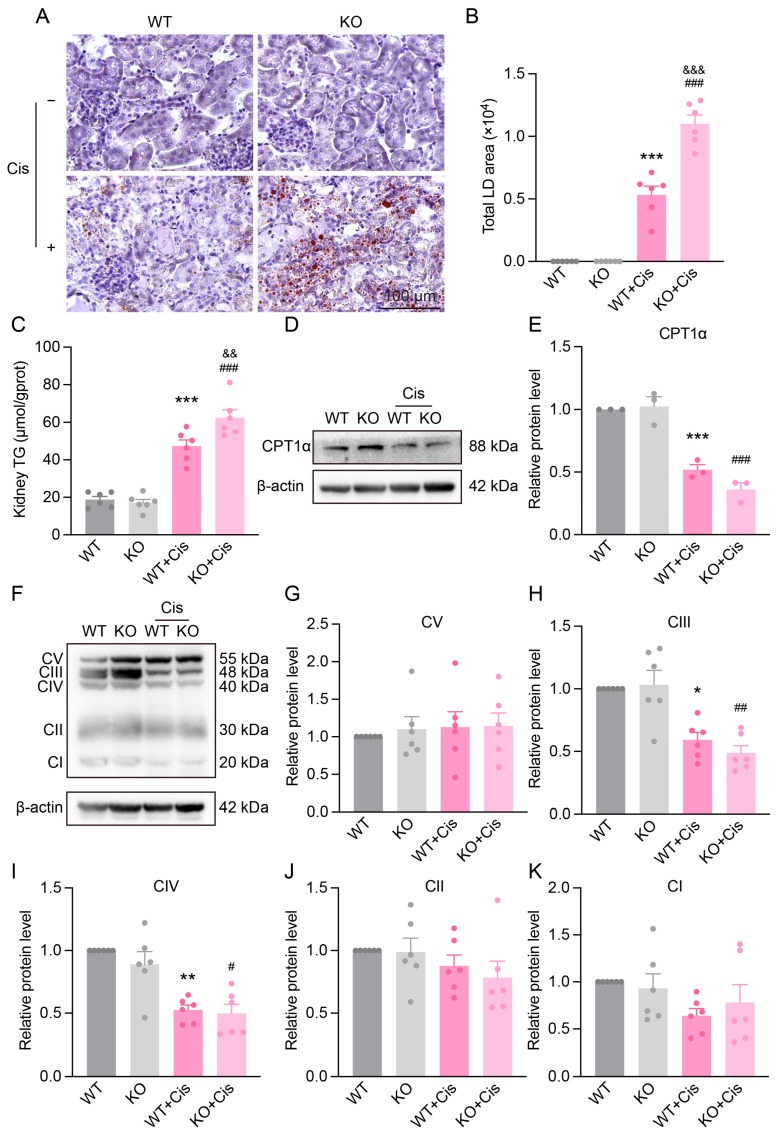
The impact of *Nfe2l2* gene knockout on cisplatin-induced metabolism disruption. (**A**) Representative Oil Red O staining images of the renal cortex in WT and KO mice three days after cisplatin treatment, scale bar = 100 µm. (**B**) Quantification of lipid droplet area from Oil Red O staining. *n* = 6. (**C**) Triglyceride levels in the renal cortex of mice with different genotypes. *n* = 6. (**D**) Representative Western blot of CPT1α in the kidneys of different genotype mice after cisplatin treatment. (**E**) Relative protein expression levels of CPT1α. *n* = 3. (**F**) Representative Western blot images of OxPhos in the kidneys of different genotype mice after cisplatin treatment. (**G**–**K**) Relative protein expression levels of OxPhos. *n* = 6. Data are presented as means ± SEM. * *p* < 0.05, ** *p* < 0.01, and *** *p* < 0.001 vs. WT group. ^#^ *p*< 0.05, ^##^ *p*< 0.01, and ^###^ *p*< 0.001 vs. KO group. ^&&^ *p* < 0.01 and ^&&&^ *p* < 0.01 vs. WT + Cis group.

To assess NRF2’s role in renal lipid metabolism, we examined lipid deposition in the kidneys of WT and KO mice. KO mice showed a significant increase in lipid droplets and higher triglyceride levels compared to WT (Figure 7A–C), indicating that NRF2 deficiency exacerbates cisplatin-induced lipid accumulation. CPT1α, a key enzyme in fatty acid oxidation, was significantly downregulated after cisplatin treatment, with a more pronounced decrease in KO mice (Figure 7D,E).

We also analyzed five key oxidative phosphorylation complexes (I–V). Cisplatin significantly reduced complex III and IV expressions in both WT and KO mice, while changes in complexes I, II, and V were mild. No significant difference was found between genotypes in the extent of reduction (Figure 7F–K).

Together with previous findings on lipid metabolism and glycolysis, these results suggest NRF2 may regulate energy metabolism by modulating early substrate conversion steps.

### 3.8. Pharmacological Activation of NRF2 Alleviates Cisplatin-Induced Renal Dysfunction and Lipid Accumulation

**Figure 8:**
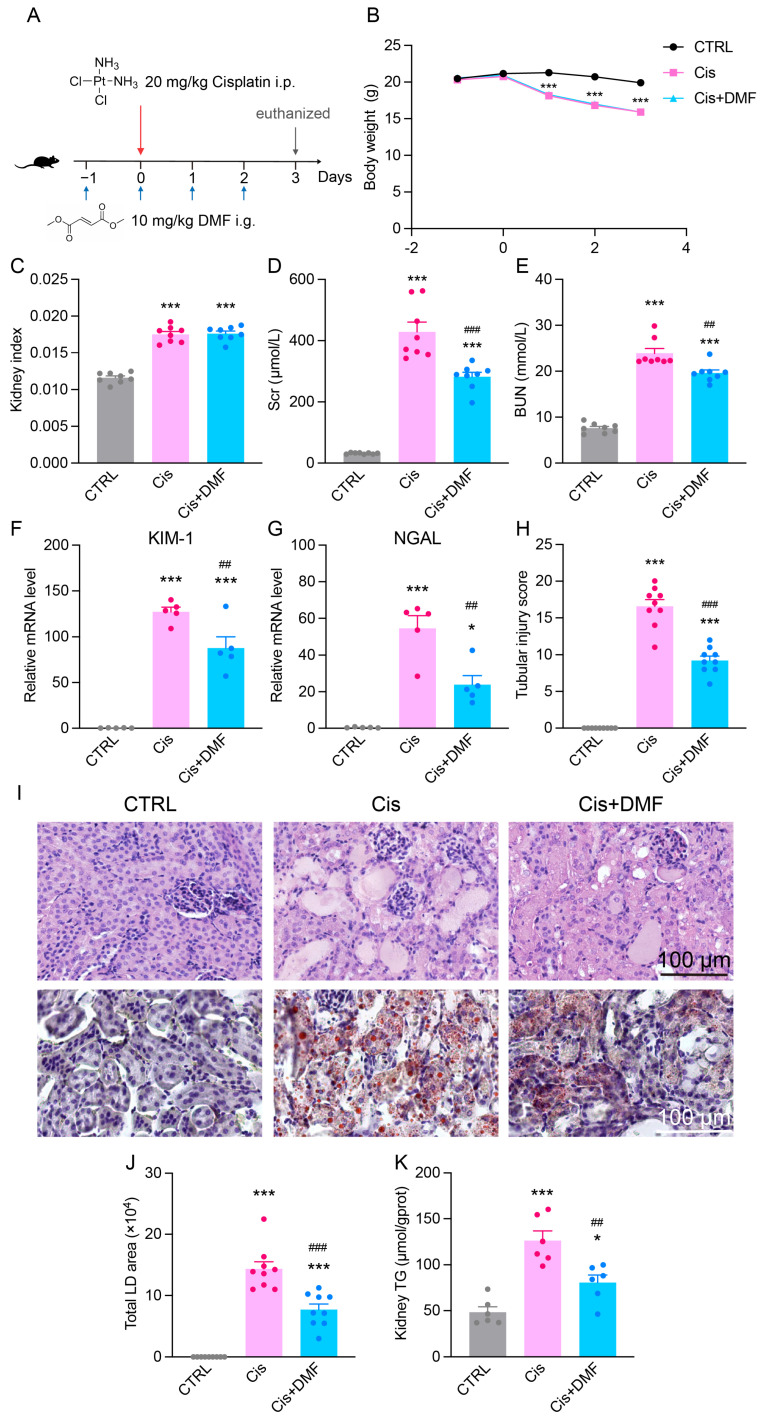
NRF2 activation alleviates renal injury and lipid deposition in Cis-AKI mice. (**A**) Schematic diagram of the DMF administration protocol. i.p.: intraperitoneal injection. i.g.: intragastric administration (**B**) Changes in mouse body weight during DMF administration. *n* = 8. (**C**) Kidney index (kidney weight/body weight) in Cis-AKI mice after DMF treatment. *n* = 8. (**D**,**E**) Scr and BUN levels in Cis-AKI mice after DMF treatment. *n* = 8. (**F**,**G**) Relative mRNA expression levels of KIM-1 and NGAL in the kidney cortex of Cis-AKI mice after DMF treatment. *n* = 5. (**H**) Quantification of tubular injury score in mouse kidney sections. *n* = 9. (**I**) Representative H&E staining and Oil Red O staining images of kidney cortex in Cis-AKI mice after DMF treatment, scale bar = 100 µm. (**J**) Quantification of lipid droplet area from Oil Red O staining. *n* = 9. (**K**) Triglyceride levels in the renal cortex of Cis-AKI mice after DMF treatment. *n* = 6. Data are presented as means ± SEM. * *p* < 0.05 and *** *p* < 0.001 vs. CTRL group. ^##^ *p*< 0.01 and ^###^ *p*< 0.001 vs. Cis group.

To evaluate the therapeutic potential of NRF2 activation, we treated Cis-AKI mice with the NRF2 activator DMF (Figure 8A). DMF significantly alleviated cisplatin-induced renal dysfunction, as shown by improved kidney function markers (Figure 8D,E). Tubular injury markers KIM-1 and NGAL were markedly reduced in DMF-treated mice (Figure 8F,G).

The histological analysis revealed that DMF decreased pathological kidney damage and tubular injury scores (Figure 8H,I). Furthermore, DMF significantly reduced renal lipid accumulation, evidenced by decreased lipid droplet area and triglyceride levels (Figure 8I–K).

## 4. Discussion

Cisplatin remains one of the most common chemotherapeutic agents associated with AKI. There is still an unmet need for effective strategies to prevent or treat Cis-AKI [6]. Therefore, a deeper understanding of the mechanisms underlying cisplatin nephrotoxicity is crucial for optimizing its clinical use. In this study, we provide evidence that NRF2 deficiency increases susceptibility to cisplatin-induced nephrotoxicity by disrupting mitochondrial homeostasis and lipid metabolism. These findings offer a theoretical basis for the development of NRF2-targeted interventions to prevent and mitigate cisplatin-induced kidney injury.

To identify key molecular players in Cis-AKI and potential therapeutic targets, we analyzed RNA sequencing data from mouse kidneys before and after cisplatin injection. KEGG and GO analyses revealed significant enrichment in metabolism-related pathways, with a focus on ROS metabolism, oxidoreductase activity, and mitochondrial components. Based on these findings, we focused on NRF2, a critical transcription factor that responds to ROS. ROS can activate NRF2 by promoting its dissociation from KEAP1 and translocation to the nucleus, where it exerts transcriptional activity [31]. Our study showed that NRF2 expression and nuclear translocation increased early after cisplatin exposure but declined with prolonged treatment. Early activation likely represents a cellular response to counteract cisplatin-induced damage, while late depletion may reflect NRF2 exhaustion. Similar dynamic NRF2 changes have been observed in other conditions like traumatic brain injury and Parkinson’s disease [32,33]. In an oxalate crystal-induced kidney injury model, NRF2 peaked 4 h post-treatment but declined by day 2, suggesting that NRF2 activation alone is insufficient to combat overwhelming oxidative stress [34]. Consistently, we also observed biphasic NRF2 expression in multiple kidney disease models. In two mouse IRI datasets, NRF2 expression increased at 4 h, peaked at 12 h, and declined thereafter [35]. In the UUO model, NRF2 expression peaked at around day 10 [36]. Our previous study on hyperuricemic nephropathy also revealed transient NRF2 activation on day 1, followed by a progressive decline [23]. Although time-resolved transcriptomic data from human kidneys are limited, proteomic and histological studies suggest that NRF2 is highly expressed in tubular epithelial cells under physiological conditions and may be ectopically upregulated in glomeruli during disease [16]. Moreover, sustained NRF2 activation has been associated with the progression from AKI to CKD in human renal tubular cells [37]. Thus, enhancing NRF2 activation exogenously may help augment antioxidant defenses and preserve organ function.

To investigate the role of NRF2 in Cis-AKI, we generated whole-body *Nfe2l2* knockout mice. Following cisplatin administration, KO mice exhibited a 50% mortality rate by day 3, with significantly elevated Scr and BUN levels, indicating increased susceptibility to Cis-AKI. However, all mice succumbed by day 4, limiting the assessment of long-term renal outcomes. In a CKD model with repeated low-dose cisplatin injections, KO mice showed a trend toward increased Scr and BUN levels, but differences were not significant, likely due to compensatory renal mechanisms. KO mice developed more severe renal fibrosis, suggesting NRF2’s protective role in CKI. The pharmacological activation of NRF2 with DMF, an NRF2 activator, starting one day before cisplatin treatment significantly attenuated kidney injury, supporting NRF2’s protective role in cisplatin nephrotoxicity.

Studies have shown that cisplatin increases mitochondrial inner membrane permeability, leading to the leakage of mitochondrial DNA into the cytoplasm, which exacerbates tubular inflammation and accelerates AKI progression [9]. Pretreatment with the mitochondria-targeted antioxidant MitoQ alleviates cisplatin-induced mitochondrial dysfunction, emphasizing the critical role of mitochondria in cisplatin nephrotoxicity, which aligns with our bioinformatic findings [38,39]. Mitochondria are the primary source of intracellular ATP, and ATP depletion is a hallmark of AKI, closely linked to poor renal outcomes [40,41]. In our study, ATP levels in the kidney declined in both WT and KO mice following cisplatin administration. However, a significant drop in ATP was observed as early as day 1 in KO mice, indicating that the absence of NRF2 accelerates mitochondrial dysfunction and impairs ATP production.

Mitochondrial dynamics—the balance between fission and fusion—is a key mechanism for maintaining mitochondrial morphology, quantity, and function [42]. FIS1 participates in mitochondrial quality control through the adaptive mitophagy pathway, and impaired mitophagy reduces the clearance of damaged mitochondria, thereby accelerating mitochondrial dysfunction [43]. It has been reported that FIS1 expression is significantly reduced in patients with focal segmental glomerulosclerosis, lupus nephritis, and CKD [44]. Mitochondrial fusion plays a protective role against AKI and subsequent fibrosis. Studies have shown that reduced MFN2 expression leads to mitochondrial damage, ROS accumulation, PTECs death, and exacerbation of renal injury [45]. Although fission and fusion are often inversely regulated under physiological conditions [46], both our present findings and previous studies have shown that the expression levels of MFN2 and FIS1 are markedly decreased in kidneys of mice with hyperuricemic nephropathy and Cis-AKI, and this downregulation is more pronounced in NRF2-deficient mice [23]. This simultaneous reduction may reflect a global dysregulation of mitochondrial dynamics under cisplatin stress, which compromises mitochondrial adaptability. NRF2 may help maintain mitochondrial function and protect the kidney from injury by regulating the balance of mitochondrial fission and fusion [31,47].

Redox imbalance is a critical early event in cisplatin-induced mitochondrial dysfunction and typically represents one of the initial cellular responses to injury [48]. In our study, cisplatin treatment led to a significant increase in NOX4 and XOD activity, alongside a marked decrease in the expression of NQO1 and GPX4, indicating that cisplatin disrupts the antioxidant defense system, thereby aggravating cellular injury. NRF2 deficiency further elevated XOD levels and reduced GPX4 expression, highlighting the essential role of NRF2 in maintaining redox homeostasis. NOX4 is a major source of ROS in the kidney, and its overactivation contributes to oxidative stress and promotes apoptosis [49]. Studies have shown that the genetic or pharmacological inhibition of NOX4 suppresses ROS production and activates the NF-*κ*B signaling pathway, thereby alleviating sepsis-induced AKI [50]. XOD generates superoxide (O_2_•^−^) through the oxidation of hypoxanthine and xanthine, contributing to ROS accumulation [51]. NQO1 and GPX4 function as key antioxidant enzymes that detoxify quinones and lipid peroxides, respectively, thus mitigating ROS-induced damage [52,53]. GPX4 is a direct transcriptional target of NRF2 and plays a pivotal role in lipid peroxidation and ferroptosis; therefore, the loss of NRF2 may aggravate renal injury by enhancing GPX4-related pathological processes [54,55]. Our findings show that cisplatin causes redox imbalance and promotes ROS accumulation, which was also validated in vitro. Excessive ROS accumulation induces oxidative damage to mitochondrial proteins, lipids, and DNA, leading to mitochondrial dysfunction and further ROS production, creating a vicious cycle [56]. Thus, through a comprehensive analysis of ATP depletion, mitochondrial dynamics, and redox imbalance, we demonstrate that cisplatin impairs mitochondrial bioenergetics, disrupts mitochondrial dynamics, and induces oxidative stress. NRF2 deficiency exacerbates these effects, ultimately resulting in significant mitochondrial dysfunction and worsened nephrotoxicity in Cis-AKI mice.

The proximal tubule, with its high energy demand, primarily relies on FAO under normal conditions. After three days of cisplatin treatment, we observed significant lipid accumulation in the kidneys, with more pronounced accumulation in *Nfe2l2* knockout mice. Previous studies have shown that NRF2 deficiency worsens the impact of a high-fat diet on the liver, increasing oxidative damage and lipid levels, highlighting NRF2’s role in metabolic regulation [57]. Ectopic lipid accumulation in non-adipose tissues leads to oxidative stress, inflammation, and apoptosis, key mechanisms in kidney disease progression [58,59]. We examined fatty acid transport and metabolic proteins to understand lipid accumulation. CD36, a key fatty acid transporter, was significantly upregulated after cisplatin treatment, indicating increased fatty acid accumulation [60,61,62,63]. However, CPT1α expression, which is essential for fatty acid transport into mitochondria for oxidation, decreased. Studies have shown that the overexpression of CPT1α in PTECs restores oxidative metabolism and reduces fibrosis [64,65]. In KO mice, CPT1α levels were further reduced, suggesting NRF2 regulates fatty acid metabolism to maintain lipid homeostasis. A study in liver-specific ACC knockout mice showed that NRF2 enhances CPT1a flux and metabolism, supporting NRF2’s role in metabolic regulation [66]. These changes in CD36 and CPT1α led to impaired fatty acid metabolism and lipid accumulation in NRF2-deficient mice, worsening energy metabolism dysfunction.

Glycolysis is the primary energy pathway during acute hypoxia, quickly generating ATP by converting glucose to lactate [67]. In AKI, cellular metabolism rapidly adjusts to meet increased energy demands. We assessed PKM2 expression, a key glycolytic enzyme, which is closely linked to the glycolysis rate. Previous studies show that activating PKM2 with TEPP-46 can prevent diabetic kidney disease by enhancing glucose metabolism, inhibiting toxic metabolites, and promoting mitochondrial biogenesis [68]. In our study, PKM2 expression increased after cisplatin treatment, along with elevated lactate and LDH levels, indicating enhanced glycolysis. Elevated LDH in hypertensive patients correlates with kidney disease progression, and increased urinary lactate may be a potential biomarker for AKI [69,70]. These changes suggest that, in Cis-AKI, kidney cells shift to glycolysis to compensate for impaired FAO, reflecting metabolic reprogramming. HIF1α, a key regulator of cellular responses to hypoxia, was also upregulated in our experiment, supporting the role of a hypoxic state in regulating kidney metabolism [71]. PKM2 can activate HIF1α, which in turn inhibits CPT1α, impairing FAO and exacerbating metabolic dysfunction [72,73]. While enhanced glycolysis supports short-term energy supply, prolonged reliance on glycolysis can cause lactate accumulation, acidosis, and further damage. The chronic suppression of FAO leads to lipotoxicity, fibrosis, and worsened kidney injury [13]. Therefore, strategies to restore FAO and metabolic balance are critical for improving recovery and preventing long-term damage after AKI.

FAO and glycolysis are the two major metabolic pathways for cellular energy substrates. They produce acetyl-CoA, which enters the TCA cycle and provides substrates and electron donors for mitochondrial oxidative phosphorylation. However, we found that the activities of electron transport chain complex III and complex IV significantly decreased after cisplatin treatment, but there were no significant changes before and after *Nfe2l2* knockout. Complex III (cytochrome bc1 complex) transfers electrons from complex I or II to cytochrome c while pumping protons into the intermembrane space, helping to form the proton gradient. Complex IV (cytochrome c oxidase) transfers electrons from cytochrome c to oxygen molecules, which are ultimately reduced to water, providing energy for ATP synthesis [74]. We speculate that the impact of NRF2 on metabolism mainly focuses on the conversion of energy substrates, with no significant effect on the final step of energy production—oxidative phosphorylation.

This study reveals the key roles of mitochondrial dysfunction, redox imbalance, and metabolic reprogramming in Cis-AKI, confirming NRF2’s protective role in the kidney, particularly through regulating mitochondrial function and lipid metabolism. However, the timing of metabolic reprogramming and potential negative effects of long-term NRF2 activation require further exploration. Our findings suggest NRF2 not only protects the kidney against antioxidative stress but also by regulating lipid metabolism, and the pharmacological activation of NRF2 effectively reduces cisplatin-induced kidney damage and lipid deposition. This study provides new insights into cisplatin nephrotoxicity and suggests NRF2 as a promising target for therapeutic strategies.

## 5. Conclusions

This study highlights the central role of mitochondrial dysfunction, redox imbalance, and metabolic reprogramming in the progression of Cis-AKI, with metabolic reprogramming potentially serving as a key early driver of disease development. We found that NRF2 is activated early in Cis-AKI, and its depletion exacerbates mitochondrial dysfunction, energy metabolic disturbances, lipid deposition, and lipotoxicity in PTECs, accelerating disease progression. The pharmacological activation of NRF2 significantly delayed the decline in kidney function and histopathological damage induced by Cis-AKI, confirming the critical protective role of NRF2-dependent antioxidant signaling. These findings not only provide insights into the molecular mechanisms underlying Cis-AKI but also offer important theoretical and experimental support for developing NRF2-based therapeutic strategies.

## Data Availability

The datasets used and analyzed during the current study are available from the corresponding author upon reasonable request.

## Abbreviations

AKI	Acute Kidney Injury
ATP	Adenosine Triphosphate
BUN	Blood Urea Nitrogen
CD36	Cluster of Differentiation 36
CKD	Chronic Kidney Disease
CK-MB	Creatine Kinase-MB Isoenzyme
Cis	Cisplatin
Cis-AKI	Cisplatin-Induced Acute Kidney Injury
CPT1α	Carnitine Palmitoyltransferase 1α
DMF	Dimethyl Fumarate
FIS1	Mitochondrial Fission 1 Protein
GEO	Gene Expression Omnibus
GO	Gene Ontology
GPX4	Glutathione Peroxidase 4
KEGG	Kyoto Encyclopedia of Genes and Genomes
KIM-1	Kidney Injury Molecule-1
LDH	Lactate Dehydrogenase
MFN2	Mitofusin 2
MS	Multiple Sclerosis
mtDNA	Mitochondrial DNA
nDNA	Nuclear DNA
NGAL	Neutrophil Gelatinase-Associated Lipocalin
NOX4	NADPH Oxidase 4
NRF2	Nuclear Factor Erythroid 2-Related Factor 2
NQO1	NAD(P)H Quinone Dehydrogenase 1
PGC1α	Peroxisome Proliferator-Activated Receptor Gamma Coactivator 1-Alpha
PKM2	Pyruvate Kinase M2
PTECs	Proximal Tubular Epithelial Cells
ROS	Reactive Oxygen Species
Scr	Serum Creatinine
XOD	Xanthine Oxidase
PKM2	Pyruvate Kinase M2

## Appendix A

**Table A1:** qPCR primer sequences.

Gene Name	Primer Sequences (5′-3′)
*Havcr1* (mouse) NM_001166631	F: ACATATCGTGGAATCACAACGAC R: ACTGCTCTTCTGATAGGTGACA
*Lcn2* (mouse) NM_008491	F: GAAATATGCACAGGTATCCTC R: GTAATTTTGAAGTATTGCTTGTTT
*Gapdh* (mouse) NM_008084	F: TGTGTCCGTCGTGGATCTGA R: TTGCTGTTGAAGTCGCAGGAG
*HAVCR1* (human) BC013325	F: TCACATCCATGTGCTGGAAT R: CGTGTGTCCTTCCGATAGGT
*LCN2* (human) NM_005564	F: GTGAGCACCAACTACAACCAGC R: GTTCCGAAGTCAGCTCCTTGGT
*ACTB* (human) NM_001101	F: CACCATTGGCAATGAGCGGTTC R: AGGTCTTTGCGGATGTCCACGT

**Table A2:** Antibodies used for Western blotting (WB), immunohistochemistry (IHC), and immunofluorescence (IF).

Name	Catalog Number	Company	WB Dilution Ratio	IHC/IF Dilution Ratio
NQO1	67240-1-Ig	Proteintech	1:10,000	/
GPX4	A11243	ABclonal	1:5000	/
NOX4	14347-1-AP	Proteintech	1:5000	/
MFN2	A12771	ABclonal	1:1000	/
FIS1	10956-1-AP	Proteintech	1:5000	/
CD36	A14714	ABclonal	1:1000	/
CD36	BD-PT-5585	Biodragon	1:2000	/
CPT1A	A5307	ABclonal	1:2500	1:200
PKM2	A18799	ABclonal	1:1000	/
HIF-1α	A11945	ABclonal	1:1000	/
NRF2	80593-1-RR	Proteintech	1:2500	/
NRF2	16396-1-AP	Proteintech	/	1:250
PGC1α	A12348	ABclonal	1:1000	/
OxPhos Rodent	45-8099	Invitrogen	1:250	/
Beta Actin	81115-1-RR	Proteintech	1:10,000	/
Lamin B1	66095-1-Ig	Proteintech	1:10,000	/
HRP-conjugated Goat Anti-Mouse IgG (H + L)	SA00001-1	Proteintech	1:10,000	/
HRP-conjugated Goat Anti-Rabbit IgG (H + L)	SA00001-2	Proteintech	1:10,000	/
Cy3-conjugated Goat Anti-Rabbit IgG (H + L)	SA00009-2	Proteintech	/	1:100
HRP-conjugated Goat Anti-Rabbit IgG (H&L)	BE0101	EASYBIO	/	1:200
